# Comparison of Clinicopathological Characteristics Between the Anterior and Posterior Type of Squamous Cell Carcinoma of the Floor of the Mouth: The Anterior Type Is a Risk Factor for Multiple Primary Cancer

**DOI:** 10.3389/fonc.2021.682428

**Published:** 2021-06-29

**Authors:** Yu Oikawa, Kae Tanaka, Toshimitsu Ohsako, Takuma Kugimoto, Takeshi Kuroshima, Hideaki Hirai, Hirofumi Tomioka, Hiroaki Shimamoto, Yasuyuki Michi, Kei Sakamoto, Tohru Ikeda, Hiroyuki Harada

**Affiliations:** ^1^ Department of Oral and Maxillofacial Surgery, Division of Oral Health Sciences, Tokyo Medical and Dental University, Tokyo, Japan; ^2^ Department of Oral Pathology, Division of Oral Health Sciences, Tokyo Medical and Dental University, Tokyo, Japan

**Keywords:** anterior type, floor of the mouth, multiple primary cancer, posterior type, squamous cell carcinoma

## Abstract

**Background:**

Floor of the mouth (FOM) squamous cell carcinoma (SCC) accounts for approximately 10% of all oral SCCs. FOM SCC can be classified into the anterior and posterior types according to their site of origin, but few studies have compared these types. This study sought to clarify differences in clinicopathological characteristics between these two types.

**Methods:**

A total of 1,220 patients with oral SCC were treated at our department from January 2001 to December 2015. Among these patients, 62 had FOM SCC. The FOM SCCs were classified into two groups: the anterior type and the posterior type. The anterior and posterior types were defined by the boundary connecting the spaces between the canine and the first premolar bilaterally. We retrospectively compared the sex, age, smoking and drinking history, clinical stage, treatment, histopathological diagnosis, multiple primary cancers, and outcomes of the two groups.

**Results:**

Among the 62 patients, 32 had the anterior type, while 30 had the posterior type. The anterior type was found more significantly in men (*p* = 0.01) and individuals with a smoking history than the posterior type (*p* = 0.04). pN2–3 cervical lymph node metastasis was significantly more common in the anterior type than in the posterior type (*p* = 0.01). The median depth of invasion in the anterior type was 4 mm. Multivariate analysis showed that the anterior type was an independent risk factor for multiple primary cancer development in FOM SCC (*p* = 0.02). The cumulative 10-year disease-specific survival rates of the anterior and posterior types were 92.8 and 95.0%, respectively, while the overall survival rates were 65.4 and 95.0%, respectively. In the anterior type FOM SCC, a lower overall survival rate was associated with multiple primary cancers and smoking-related diseases.

**Conclusion:**

Smoking cessation and adequate systemic screening for multiple primary cancers are needed to improve the prognosis of FOM SCC, particularly the anterior type.

## Introduction

The floor of the mouth (FOM) is a small, horseshoe-shaped region situated beneath the movable part of the tongue and above the muscular diaphragm formed by the mylohyoid muscles (1). The abundant lymphatic vessels in this region frequently cause lymphogenous spread of FOM cancer, including providing a direct pathway to the mid-internal jugular and/or contralateral nodes (2), thereby greatly increasing the need for more complex treatment. Therefore, a comprehensive assessment of tumor invasion into the surrounding tissues, appropriate surgical margins, and neck dissection (ND) strategies are necessary.

FOM cancers are uncommon, accounting for only 10% of all oral cancers in Japan (3). In previous studies, FOM cancers were analyzed as a subset of all oral cancers (4) or concomitantly with tongue cancer (5). There are several important differences between FOM and other subsites of oral cancers (2). One important difference is the considerable anatomical complexity of FOM cancers compared to that in other subsites. Another notable difference is the role of tobacco smoking and alcohol consumption as significant risk factors for FOM cancer. Their conferred risk of FOM cancer is much more than that of other subsites (6). Moreover, FOM cancer is more prone to have cervical lymph node metastases than tongue cancers even with similar thickness (5). Furthermore, the incidence of multiple primary cancers in patients with FOM squamous cell carcinoma (SCC) is higher than that of tongue SCC (7). These findings are only some of the specific properties of FOM cancer.

Based on the location of the primary tumor, FOM cancer is divided into the anterior and posterior types (8). Anatomically, the two types have different characteristics. The posterior type is closer to the oropharynx, while the anterior type is the area over the geniohyoid muscle, where lymphatics flow contralaterally. Despite the clear anatomical distinction between the anterior and posterior types of FOM cancer, no comparative analysis of these types has been reported.

The purpose of this study was to determine the clinicopathological differences between the anterior and posterior types of FOM SCC.

## Materials and Methods

### Patients

We retrospectively reviewed the medical records of 1,220 patients with primary oral SCC treated at the Department of Oral and Maxillofacial Surgery, Tokyo Medical and Dental University, Japan, between January 2001 and December 2015. Among these patients, 77 had FOM SCC. A total of 15 patients were excluded from this study, two of whom had multiple primary SCC in the oral cavity, while the remaining 13 were treated with brachytherapy. Finally, 62 patients of FOM SCC who underwent radical surgical treatment of their primary tumor were enrolled for this study. The observation period was set from the date of the initial visit to our department until December 31, 2020, and the mean follow-up period was 101.1 months (range, 3.5–223.5 months). The definitions of the anterior and posterior types of FOM SCC were based on the recommendations of the Japanese Society of Oral Oncology (9). Specifically, the line connecting the spaces between the canine and the first premolar bilaterally was defined as the boundary between the anterior and posterior FOM areas.

The study was conducted in accordance with the 1964 Declaration of Helsinki and its latest amendments. Ethical approval was granted by the institutional review board of Tokyo Medical and Dental University, Faculty of Dentistry (No. D2015-600). Written informed consent was obtained from all patients.

### Variables

The following information was collected from the patients’ medical records: sex, age, smoking and drinking history, clinical stage, treatment, histopathological diagnosis of the primary tumor and cervical lymph node, presence of multiple primary cancers, and outcomes. To estimate the cumulative dose of smoking, we used the Brinkman index, which was calculated by multiplying the number of cigarettes smoked per day by the number of years of smoking. For alcohol consumption, the Sake index, which was calculated by multiplying the number of glasses (180 ml/glass) of sake per day by the number of years of drinking, was used. Information on smoking and drinking history was available for the 28 patients with the anterior type and 26 patients with the posterior type. The clinical stage of the tumor was classified based on the 8th edition of the TNM Classification defined by the Union for International Cancer Control (UICC).

In the treatment of the tumor of primary site, the resected surrounding tissue, histopathological invasion, and pathological depth of invasion (DOI) were examined in all patients. ND was performed in 39 patients, in whom the following were investigated: surgical procedure, laterality, pathological nodal status, metastatic level, and the relationship between their pN status and the DOI of their primary tumor site. Adjuvant therapy was performed in 12 patients, and their treatment regimens were reviewed. In patients with multiple primary cancers, the site and time of occurrence were examined. The Warren and Gates’s criteria (10) was used to diagnose multiple primary cancers. Multiple primary cancers that were diagnosed within a period of less than one year were defined as synchronous cancers, while those that were diagnosed at an interval of one year or longer were defined as heterochronous cancers (9).

The survival rates in the anterior and posterior types of FOM SCC were calculated as disease-specific survival (DSS) and overall survival (OS). DSS was defined as the period from the date of the initial visit to our department until death from the FOM SCC or the last follow-up date. OS was defined as the period from the first date of visiting our department until death or last follow-up date.

### Statistical Analysis

All statistical analyses were performed with EZR version 1.41 (Saitama Medical Center, Jichi Medical University, Saitama, Japan) (11), which is a graphical user interface for R version 3.6.2 (The R Foundation for Statistical Computing, Vienna, Austria). Continuous variables were analyzed using the Mann–Whitney U test and Kruskal–Wallis test. Fisher’s exact probability test was used for categorical data. The Jonckheere–Terpstra test was used to analyze the trend of continuous variables. Multivariate analysis was performed using logistic regression analysis. A receiver operating characteristic (ROC) curve was used to identify a possible cutoff value of DOI that could serve as an optimal predictor for cervical lymph node metastasis. The Kaplan–Meier method was adopted to calculate DSS and OS rates of the two types, which were compared using the log-rank test. Two-tailed *p*-values <0.05 were considered statistically significant.

## Results

### General Characteristics

Among the 62 patients, 32 had the anterior type (median age 65.5 years; 31 men; one woman), while 30 had the posterior type (median age 62.5 years; 22 men; eight women) (**Table 1**). A male predominance was observed in the anterior type than in the posterior type (*p* = 0.01). The amount of smoking was significantly higher in patients with the anterior type than in those with the posterior type (*p* = 0.04). On the other hand, there were no significant differences in alcohol consumption and UICC stage between the two groups (*p* = 0.60, *p* = 0.64, respectively). Half of the patients in each type showed a histologically well-differentiated tumor, while only rare cases had a poorly-differentiated tumor.

**Table 1:** Patients characteristics by subsite of floor of the mouth.

	Anterior type	Posterior type	*p* value
	(*N* = 32)	(*N* = 30)	
Sex, *N* (%)			
Male	31 (96.9)	22 (73.3)	0.01
Female	1 (3.1)	8 (26.7)	
Age (years)			
Median (range)	65.5 (31–84)	62.5 (29–77)	0.17
Brinkman index			
Median (range)	920 (0–2000)	500 (0–2280)	0.04
Sake index			
Median (range)	76.5 (0–462)	76.0 (0–285)	0.60
UICC stage, *N* (%)			
I	8 (25.0)	10 (33.3)	0.64
II	13 (40.6)	14 (46.7)	
III	5 (15.6)	2 (6.7)	
IVA	6 (18.8)	4 (13.3)	
Histological differentiation, *N* (%)			
Well	16 (50.0)	15 (50.0)	1.00
Moderately	14 (43.8)	13 (43.3)	
Poorly	2 (6.2)	2 (6.7)	
Neck dissection, *N* (%)			
Not performed	13 (40.6)	10 (33.3)	0.19
Elective neck dissection	7 (21.9)	14 (46.7)	
Therapeutic neck dissection	9 (28.1)	4 (13.3)	
Subsequent neck dissection	3 (9.4)	2 (6.7)	

UICC, Union for International Cancer Control.

### Treatment of the Primary Tumor

We performed surgical resection of the primary tumor in all cases. In both types, a combined resection involving the tongue and mandible was the most common (**Table 2**). Tumor invasion into the surrounding tissue was pathologically identified in 23 patients with the anterior type and 19 patients with the posterior types, respectively. In these cases, genioglossus invasion was more often recognized in the anterior than in the posterior type (*p* = 0.04). There were no patients with geniohyoid muscle involvement. The median DOI of the anterior and posterior types were 4.0 and 1.8 mm, respectively (*p* = 0.24).

**Table 2:** Surgical resection and tumor invasion.

	Anterior type	Posterior type	*p* value
	(*N* = 32)	(*N* = 30)	
Resected tissue, *N* (%)			
FOM only	9 (28.1)	10 (33.3)	0.73
FOM + T	6 (18.8)	7 (23.3)	
FOM + M	1 (3.1)	2 (6.7)	
FOM + T + M	16 (50.0)	11 (36.7)	
Tumor invasion into the surrounding tissue, *N* (%)			
Sublingual gland	22 (95.7)	16 (84.2)	0.30
Intrinsic muscle of tongue	14 (60.9)	13 (68.4)	1.00
Genioglossus muscle	12 (52.2)	4 (21.1)	0.04
Mandible	4 (17.4)	1 (5.3)	0.36
DOI (mm)			
Median (range)	4.0 (0.1–22.0)	1.8 (0.1–16)	0.24

FOM, floor of the mouth; T, tongue; M, mandible; DOI, depth of invasion.

### Treatment of the Neck

Overall, 16 patients with the anterior type and 18 with the posterior type underwent ND at initial therapy. We also performed subsequent ND in three patients with the anterior type and two with the posterior type cases. Our strategy was to perform modified radical ND in cases that were clinically N-positive, and supraomohyoid or suprahyoid ND in cases that were clinically N-negative and/or requiring reconstruction using a free flap. The laterality of ND was as follows: 14 bilateral and five ipsilateral in patients with the anterior types, and two bilateral and 18 ipsilateral in those with the posterior type. Histopathologically, there were confirmed cervical lymph node metastases in 14 patients with the anterior type and six with the posterior type. In those with the anterior type, there were one, four, six, and three cases with pN2a, pN2b, pN2c, and pN3b stages, respectively, while in those with the posterior type, there were two, one, and three cases with pN1, pN2c, and pN3b stages, respectively. Lymph node metastasis status categorized as pN0–1 and pN2–3 was analyzed to revealed that pN2–3 was significantly more common in the anterior type (*p* = 0.01) (**Table 3**). The most common metastasis level was level I in the anterior type (85.7%) and level III in the posterior type (66.7%). In patients with the anterior type and bilateral lymph node metastases, the primary tumor crossed the midline in seven out of eight cases. No cervical lymph node metastasis was observed in patients with no surrounding tissue invasion or sublingual gland invasion. On the other hand, of the 16 patients with the anterior type and of the 13 with the posterior type who had an invasion of the other surrounding tissues, neck metastases were confirmed in 14 patients with the anterior type and six with the posterior type.

**Table 3:** Relationship between pathological N stage and DOI.

	Anterior type	Posterior type	*p* value
	(*N* = 32)	(*N* = 30)	
Pathological N stage, *N* (%)			
pN0–1	18 (56.3)	26 (86.7)	0.01
pN2–3	14 (43.7)	4 (13.3)	
DOI (mm), median (range)			
pN0–1	1.5 (0.1–14)	1.1 (0.1–14)	<0.01
pN2–3	8.0 (0.1–22)	5.0 (1.5–16)	

DOI, depth of invasion.

**Figure 1:**
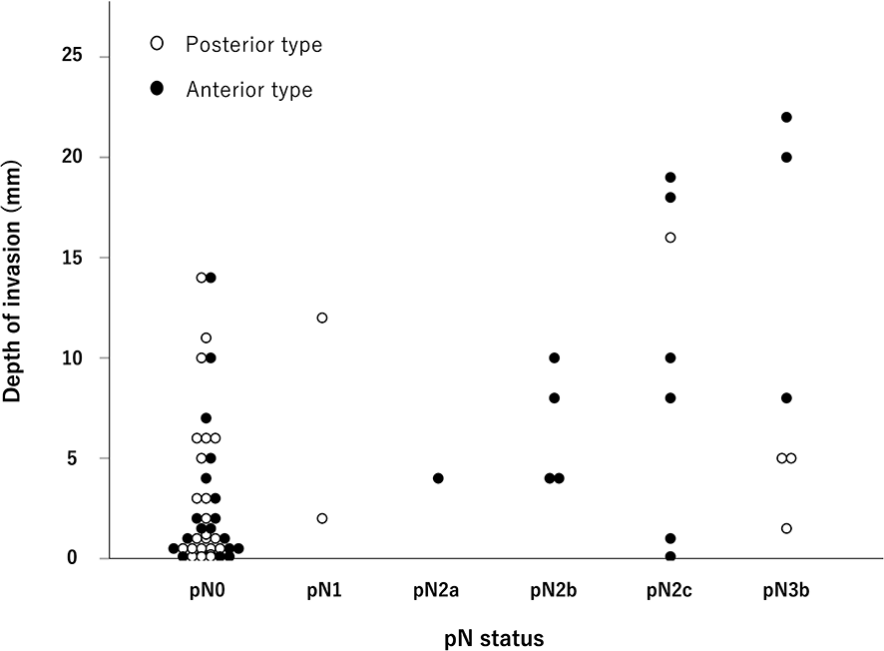
Distribution of the pathological N stage and depth of invasion. As the pathological N stage progressed, the depth of invasion also tended to increase, especially for the anterior type (*p <* 0.01, Jonckheere–Terpstra test).

The DOI for each pN status is shown in **Figure 1**. In the analysis of all 62 cases, there was a trend for the DOI to increase as the pN stage progressed (*p <*0.01). This trend was also observed in the anterior type (*p <*0.01). The values of the median DOI for anterior/pN0–1, anterior/pN2–3, posterior/pN0–1, and posterior/pN2–3 were 1.5, 8.0, 1.1, and 5.0 mm, respectively (*p <*0.01) (**Table 3**). In the anterior type, we investigated the optimal DOI cut-off point between pN0–1 and pN2–3 using ROC analysis, and found that an 4 mm threshold yielded a sensitivity of 85.7%, specificity of 72.2%, and area under the curve of 0.802 [95% confidence interval (CI) = 0.636–0.967].

### Adjuvant Therapy

Adjuvant therapy was performed in 12 patients. We conducted postoperative adjuvant therapy for patients with the following conditions (1): primary tumors near the resection margin, (2) primary tumors with pathologically confirmed extensive invasion of the surrounding tissue, (3) pathological metastatic lymph nodes ≥4 at the neck, and/or (4) extranodal extension with adhesion to surrounding tissue (12). The median radiation dose was 50 Gy (range, 40–50 Gy). This was based on the results of a study conducted at our institution, which reported that there was no difference in the control rate between postoperative adjuvant therapy doses of 50 and 66 Gy (12). Four patients received radiotherapy (50 Gy) to the primary site, and one of them received S-1, which is an oral fluorouracil antitumor drug that combines tegafur, gimeracil, and oteracil. Two patients underwent radiotherapy (50 Gy) to the neck, one of whom received platinum-based anticancer agents, while the other received S-1. Six patients received radiotherapy (40–50 Gy) to the primary site and the neck, one of whom received platinum-based anti-cancer agents, while the other one received S-1. Patients with poor general status and/or decline of renal function did not receive concomitant chemotherapy.

### Multiple Primary Cancer and the Risk Factor

Computed tomography (CT), magnetic resonance imaging (MRI), and positron emission tomography (PET) were performed before initial treatment. Screening of the upper gastrointestinal tract by endoscopy was performed in the perioperative period, and CT was also ordered one or two times per year for at least two years after the primary treatment. Multiple primary cancers were detected in 53.1% (17/32) of the patients with the anterior type and 16.7% (5/30) of the posterior type (*p <*0.01), for a total of 28 sites (**Table 4**). All patients were men. Among the 28 sites with multiple primary cancers, nine were synchronous, while 19 were involved heterochronous. The esophagus was the most common site (*N* = 9), followed by the stomach (*N* = 5), larynx (*N* = 3), and large intestine (*N* = 3). Uni- and multivariate analysis of risk factors for multiple primary cancers were conducted, limiting analysis to male. The anterior type was identified as a significant independent risk factor [odds ratio = 5.03, 95% CI = 1.28–19.7, *p* = 0.02 (**Table 5**)].

**Table 4:** Multiple primary cancers of the case with FOM SCC.

	Anterior type	Posterior type	*p* value
	(*N* = 32)	(*N* = 30)	
The number of additional primary cancer, *N*			
1	13	4	
2	4	0	
3	0	1	
Total, N (%)	17 (53.1)	5 (16.7)	<0.01

FOM, floor of the mouth; SCC, squamous cell carcinoma.

**Table 5:** Risk factors for multi primary cancers in males (*N* = 53).

Univariate analysis		
	Incidence rate (%)	*p* value
Age		
≥60 years	45.9	0.38
<60 years	31.3	
Brinkman index		
≥600	v34.4	0.25
<600	52.6	
Sake index		
≥60	35.5	0.76
<60	41.2	
Site		
Anterior	54.8	0.03
Posterior	22.7	
**Multivariate analysis**		
	**Odd ratio (95% CI)**	***p* value**
Brinkman index		
≥600	0.31 (0.08–1.17)	0.08
<600	1.00 (ref)	
Site		
Anterior	5.03 (1.28–19.7)	0.02
Posterior	1.00 (ref)	

### Treatment Outcome

Primary recurrence occurred in two patients with the anterior type and one patient with the posterior type. One case of each type was rescued by surgery, while the other patient with the anterior type could not be controlled even with proton beam therapy. Neck recurrence was observed in one patient with the anterior type and one with the posterior type. In both cases, surgery was performed. The anterior type case died of neck failure. Local control and locoregional control rates were 96.9% (31/32) and 93.8% (30/32), respectively, for the anterior type and 100% for both rates for the posterior type.

**Figure 2:**
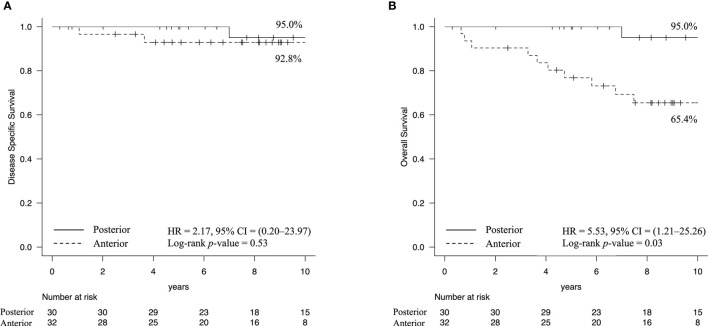
Kaplan–Meier estimates of survival rate. **(A)** The cumulative 10-year disease-specific survival rates were 92.8% in the anterior type and 95.0% in the posterior type. **(B)** In contrast, the overall survival rates were 65.4% in the anterior type and 95.0% in the posterior type (Log-rank *p*-value = 0.03). These results indicated that patients with the anterior type had a higher likelihood of death due to other diseases.

The cumulative 10-year DSS rates were 92.8% in the anterior type and 95.0% in the posterior type [hazard ratio [HR] = 2.17, 95% CI = 0.20–23.97, Log-rank *p*-value = 0.53 (**Figure 2A**)], while the cumulative 10-year OS rates were 65.4% in the anterior type and 95.0% in the posterior type [HR = 5.53, 95% CI = 1.21–25.26, Log-rank *p*-value = 0.03 (**Figure 2B**)]. The cause of death in the posterior type was the primary disease in one case, while in the anterior type, death was due to the primary disease in two cases, death due to other cancers in three cases, and death due to intercurrent disease, including heat-related illnesses and lung diseases, in five cases.

## Discussion

Our study revealed some characteristic differences between the anterior and posterior types of FOM SCC. The distribution of the two types was almost equal. The incidence of the anterior types was higher in individuals with a smoking history than that of the posterior type. pN2–3 was significantly more common in the anterior type than that in the posterior type, and the median depth of invasion in these cases was 4 mm. Multivariate analysis showed that the anterior type was an independent risk factor for the development of multiple primary cancers in patients with FOM SCC (*p* = 0.02). The cumulative 10-year disease-specific survival rates in the anterior and posterior types were 92.8 and 95.0%, respectively, while the overall survival rates were 65.4 and 95.0%, respectively.

Feind and Cole (8) subdivided the FOM into two areas, anterior and posterior to a line connecting the spaces between the first molar and the second molar on the left and on the right side. In their report, 80% of the cases were of the anterior type (8). The Japanese Society of Oral Oncology guidelines defines an equivalent site between the canine and the first premolar as the boundary between the anterior and the posterior regions (9), which is a more anterior location compared with the boundary set by Feind and Cole (8). This may explain why our proportion of anterior type disease was lower than in their study (51.6% *vs.* 80%).

Advances in gene mutation analysis technology have revealed that the overall picture of human cancer mutations can be divided into 21 features, termed “Mutational Signatures”, by analysis that classifies the base substitution patterns of mutations and clarifies the biological process of mutation and related background factors (13). In this mutational signature analysis, tobacco-induced base substitution patterns are classified as “Signature 4”, which is an imprint of the bulky DNA adducts and their removal, and resulting in mutation of C > A, including lung adenocarcinoma, SCC, small cell carcinomas, head and neck SCC, and liver cancer (13). Furthermore, smoking has been shown to elevate the mutation burden with “Signature 5” in oral cancer (14). “Signature 5” mutation is likely associated with age at diagnosis of the cancer, suggesting the relationship between “Signature 5” mutation and the cumulative effect of DNA damage and repair in progenitor cells as an oncogenic mechanism rather than direct DNA damage by smoke carcinogens (15). Another mechanism to induce oral cancer could involve a synergistic effect of smoking and alcohol consumption, given that their combination increased the relative risk of head and neck SCC by 15-fold, although alcohol could also be a single risk factor (15–17). In Japan, people with Brinkman index scores >200 are advised to visit a smoking cessation clinic as the lung cancer risk is particularly high for Brinkman index scores >400–600. After smoking cessation, the risks for oral cavity and oropharyngeal SCC decline over time, approaching that of non-smokers after ≥10 years (16); thus, education for preventing smoking initiation is also necessary. It has been reported that oral cancer develops in men with a Sake index ≥60 and in women with an even with less consumption of alcohol (Sake index = 1–59) (18), suggesting potential sex differences in the effects of alcohol on oral cancer development. In our study, the anterior type had a significantly higher Brinkman index than that in the posterior type, which may be due to anatomical features that make the anterior part of the FOM more vulnerable to exposure to cigarette smoke, which contains carcinogens.

Steinhart et al. (19) showed that 90% of FOM cancers invaded the surrounding tissues: invasion of the sublingual gland, intrinsic muscles of the tongue, genioglossus muscle, and mandibular bone accounted for 93, 65, 28, and 12% of such cases, respectively. Our histopathological analysis showed invasion of the surrounding tissues in 67.7% (42/62) of cases. Due to the anatomical location, FOM SCC can easily invade the surrounding tissues; thus, appropriate resection based on the preoperative diagnosis is necessary during surgery.

A previous study reported that FOM cancers with a thickness of 2.1–4 mm had higher rates of metastasis than those in tongue cancers of the same thickness (5). Based on these observations, elective ND would be appropriate for all FOM cancers with a thickness >2 mm (5). In our study, we found that the anterior type had significantly more pN2–3 than that in the posterior type, and the optimal DOI cutoff value for pN2–3 in the anterior type was 4 mm. This may assist in the selection of the extent of ND and postoperative adjuvant therapy, and in the prediction of cervical lymph node metastasis.

The incidence of multiple primary cancers in patients with oral cancer is reportedly 0.73–7.22% (20) and that of FOM is 10–36% (20, 21). Ariji et al. (20) stated that FOM cancer was less likely to be affected by chronic stimulation from teeth or prostheses than tongue cancer, even though FOM is a continuation of the tissue of the tongue. This means that FOM cancers tend to reflect the concept of field cancerization other than by direct mechanical stimulation, inducing multiple organ cancer associated with field cancerization. In our department, we treated 1,220 cases with oral SCC between 2001 and 2012, among which 167 cases (13.7%) presented with multiple primary cancers (data not shown). All cases of multiple primary cancers developed in men. Multiple primary cancers were observed in 35.5% of all patients with FOM SCC and 53.1% of patients with anterior type FOM SCC. Our analysis revealed that the anterior type is an independent risk factor for multiple primary cancers with FOM SCC. For early detection of multiple cancers, careful screening, including endoscopy and PET, in addition to CT and MRI, is important for FOM SCC, particularly the anterior type.

The DSS rates for the anterior and posterior types were favorable: 92.8 and 94.7%, respectively. Our analysis demonstrated that the OS rate for the anterior type was significantly low because of the number of patients who died of other cancers or causes, including smoking-related diseases. Sessions et al. (21) reported that 53% of FOM cancer cases with multiple primary cancers died of other cancers. The control of the second cancer is likely more difficult than that of primary cancer (20); thus, early detection and treatment of multiple primary cancers are important to improve the OS, particularly in the anterior type FOM SCC.

Our retrospective and exploratory research study had some limitations. Notably, the cohort was very small (*N* = 62) because this was a single-center study. Additionally, a large number of predictors were analyzed relative to the small number of patients; therefore, the results need to be carefully interpreted. In the future, further prospective studies are necessary with a large number of cases in multicenter institutions as well as elucidating the molecular biology supporting the clinical condition using surgical specimens.

## Conclusion

These results demonstrate different clinicopathological features between the anterior and posterior type of FOM SCCs. Compared with the posterior type, the anterior type was associated with smoking and more cases of advanced N stage. Furthermore, the anterior type was an independent risk factor for the development of multiple primary cancers in patients with FOM SCC. A favorable DSS for FOM SCC can be expected with appropriate surgical treatment based on preoperative diagnosis of tumor invasion and metastatic lymph nodes. To improve the OS, particularly in patients with anterior type FOM SCC, smoking cessation is necessary. In addition, adequate follow-up using multiple modalities, including gastroscopy or PET, is recommended for the early detection of multiple primary cancers.

## Data Availability Statement

The original contributions presented in the study are included in the article/supplementary material. Further inquiries can be directed to the corresponding author.

## Ethics Statement

The studies involving human participants were reviewed and approved by the institutional review board of Tokyo Medical and Dental University, Faculty of Dentistry. The patients/participants provided their written informed consent to participate in this study.

## Author Contributions

YO, KT, and HHa conceptualized this study. YO, KT, TO, TKug, TKur, HHi, and HT curated and investigated the data. YO and KT conducted the formal analysis. KS and TI performed the pathological investigation. HS, YM, and HHa supervised and administrated this study. YO and KT wrote the draft of the manuscript, and YO and HHa reviewed and edited the manuscript. All authors contributed to the article and approved the submitted version.

## Conflict of Interest

The authors declare that the research was conducted in the absence of any commercial or financial relationships that could be construed as a potential conflict of interest.

## Abbreviations

CT, computed tomography; DOI, depth of invasion; DSS, disease specific survival; FOM, floor of the mouth; MRI, magnetic resonance imaging; ND, neck dissection; OS, overall survival; PET, positron emission tomography; ROC, receiver operating characteristic; SCC, squamous cell carcinoma.

## References

[1] Standring S. Gray’s Anatomy: The Anatomical Basis of Clinical Practice. 1st ed. Philadelphia: Elsevier Limite (2016).

[2] Shaw HJ Hardingham M. Cancer of the Floor of the Mouth: Surgical Management. J Laryngol Otol (1977) 91(6):467–88. 10.1017/s002221510008395x 327001

[3] Ariyoshi Y Shimahara M Omura K Yamamoto E Mizuki H Chiba H. Epidemiological Study of Malignant Tumors in the Oral and Maxillofacial Region: Survey of Member Institutions of the Japanese Society of Oral and Maxillofacial Surgeons, 2002. Int J Clin Oncol (2008) 13(3):220–8. 10.1007/s10147-007-0756-9 18553231

[4] Radoï L Paget-Bailly S Cyr D Papadopoulos A Guida F Schmaus A. Tobacco Smoking, Alcohol Drinking and Risk of Oral Cavity Cancer by Subsite: Results of a French Population-Based Case-Control Study, the ICARE Study. Eur J Cancer Prev (2013) 22(3):268–76. 10.1097/CEJ.0b013e3283592cce 22976386

[5] Balasubramanian D Ebrahimi A Gupta R Gao K Elliott M Palme CE. Tumour Thickness as a Predictor of Nodal Metastases in Oral Cancer: Comparison Between Tongue and Floor of Mouth Subsites. Oral Oncol (2014) 50(12):1165–8. 10.1016/j.oraloncology.2014.09.012 25307875

[6] Hashibe M Brennan P Chuang SC Boccia S Castellsague X Chen C. Interaction Between Tobacco and Alcohol Use and the Risk of Head and Neck Cancer: Pooled Analysis in the International Head and Neck Cancer Epidemiology Consortium. Cancer Epidemiol Biomarkers Prev (2009) 18(2):541–50. 10.1158/1055-9965.EPI-08-0347 PMC3051410 19190158

[7] Gilbert EH Goffinet DR Bagshaw MA. Carcinoma of the Oral Tongue and Floor of Mouth: Fifteen Years’ Experience With Linear Acceleration Therapy. Cancer (1975) 35(6):1517–24. 10.1002/1097-0142(197506)35:6<1517::aid-cncr2820350607>3.0.co;2-3 807312

[8] Feind CR Cole RM. Cancer of the Floor of the Mouth and Its Lymphatic Spread. Am J Surg (1968) 116(4):482–6. 10.1016/0002-9610(68)90378-4 5676896

[9] Izumo T Kirita T Ariji E Ozeki S Okada N Okabe S. General Rules for Clinical and Pathological Studies on Oral Cancer: A Synopsis. Jpn J Clin Oncol (2012) 42(11):1099–109. 10.1093/jjco/hys141 23024282

[10] Warren S Gates O. Multiple Primary Malignant Tumors. A Survey of the Literature and a Statistical Study. Am J Cancer (1932) 16:1358–414.

[11] Kanda Y. Investigation of the Freely Available Easy-to-Use Software ‘Ezr’ for Medical Statistics. Bone Marrow Transplant (2013) 48(3):452–8. 10.1038/bmt.2012.244 PMC3590441 23208313

[12] Hirai H Ohsako T Kugimoto T Tomioka H Michi Y Kayamori K. Comparison of 50- and 66-Gy Total Irradiation Doses for Postoperative Cervical Treatment of Patients With Oral Squamous Cell Carcinoma. Oral Oncol (2020) 107:104708. 10.1016/j.oraloncology.2020.104708 32339995

[13] Alexandrov LB Nik-Zainal S Wedge DC Aparicio SA Behjati S Biankin AV. Signatures of Mutational Processes in Human Cancer. Nature (2013) 500(7463):415–21. 10.1038/nature12477 PMC3776390 23945592

[14] Alexandrov LB Ju YS Haase K Van Loo P Martincorena I Nik-Zainal S. Mutational Signatures Associated With Tobacco Smoking in Human Cancer. Science (2016) 354(6312):618–22. 10.1126/science.aag0299 PMC6141049 27811275

[15] Bradley G Magalhaes MA Hyrcza M. Mutational Signatures in Oral Cancer Indicate a Complex Role for Tobacco Smoke Carcinogens. Oral Dis (2018) 24(5):682–4. 10.1111/odi.12665 28295873

[16] Chi AC Day TA Neville BW. Oral Cavity and Oropharyngeal Squamous Cell Carcinoma–an Update. CA Cancer J Clin (2015) 65(5):401–21. 10.3322/caac.21293 26215712

[17] Turati F Garavello W Tramacere I Pelucchi C Galeone C Bagnardi V. A Meta-Analysis of Alcohol Drinking and Oral and Pharyngeal Cancers: Results From Subgroup Analyses. Alcohol Alcohol (2013) 48(1):107–18. 10.1093/alcalc/ags100 22949102

[18] Nomura T Shibahara T Noma H Yamane G Yokoyama A Muramatsu T. A Study of Smoking and Drinking Habits as Carcinogens in the Development of Oral Cancer. Jpn Soc Head Neck Cancer (1998) 24:83–9. 10.5981/jjhnc1974.24.83

[19] Steinhart H Kleinsasser O. Growth and Spread of Squamous Cell Carcinoma of the Floor of the Mouth. Eur Arch Otorhinolaryngol (1993) 250(6):358–61. 10.1007/BF00188386 8260147

[20] Ariji E Jingu K Motooka M Masuda K. Multiple Primary Cancers Arising in Patients With Cancer of the Oral Floor. J Jpn Stomatol Soc (1988) 37:654–9.

[21] Sessions DG Spector GJ Lenox J Parriott S Haughey B Chao C. Analysis of Treatment Results for Floor-of-Mouth Cancer. Laryngoscope (2000) 110(10 Pt 1):1764–72. 10.1097/00005537-200010000-00038 11037841

